# Adapting Single-Image Super-Resolution Models to Video Super-Resolution: A Plug-and-Play Approach

**DOI:** 10.3390/s23115030

**Published:** 2023-05-24

**Authors:** Wenhao Wang, Zhenbing Liu, Haoxiang Lu, Rushi Lan, Yingxin Huang

**Affiliations:** School of Computer Science and Information Security, Guilin University of Electronic Technology, Guilin 541004, China

**Keywords:** video super-resolution, single-image super-resolution, plug-and-play, deformable convolution

## Abstract

The quality of videos varies due to the different capabilities of sensors. Video super-resolution (VSR) is a technology that improves the quality of captured video. However, the development of a VSR model is very costly. In this paper, we present a novel approach for adapting single-image super-resolution (SISR) models to the VSR task. To achieve this, we first summarize a common architecture of SISR models and perform a formal analysis of adaptation. Then, we propose an adaptation method that incorporates a plug-and-play temporal feature extraction module into existing SISR models. The proposed temporal feature extraction module consists of three submodules: offset estimation, spatial aggregation, and temporal aggregation. In the spatial aggregation submodule, the features obtained from the SISR model are aligned to the center frame based on the offset estimation results. The aligned features are fused in the temporal aggregation submodule. Finally, the fused temporal feature is fed to the SISR model for reconstruction. To evaluate the effectiveness of our method, we adapt five representative SISR models and evaluate these models on two popular benchmarks. The experiment results show the proposed method is effective on different SISR models. In particular, on the Vid4 benchmark, the VSR-adapted models achieve at least 1.26 dB and 0.067 improvement over the original SISR models in terms of PSNR and SSIM metrics, respectively. Additionally, these VSR-adapted models achieve better performance than the state-of-the-art VSR models.

## 1. Introduction

Numerous videos are captured every day; however, due to the different capabilities of sensors, the quality of captured videos can vary greatly, which affects the subsequent analysis and applications [1,2,3,4]. Recently, computer technologies have been applied to many fields [5,6,7,8]. In particular, video super-resolution (VSR) is a technology for improving the quality of captured video. It produces high-resolution (HR) video frames from their low-resolution (LR) counterparts. The VSR problem is challenging due to its ill-posed nature, but its applications include video display, video surveillance, video conferencing, and entertainment [9].

VSR models take consecutive frames as input. Single-image super-resolution (SISR) methods process only one image at a time. So, VSR models take both spatial information and temporal information into account, while SISR models only exploit spatial information for super-resolution (SR) reconstruction. Thus, many VSR methods adapt SISR models for spatial information extraction. For example, Haris et al. [10] introduced RBPN, which employs blocks from DBPN [11] in a recurrent encoder–decoder module to utilize spatial and temporal information. Tian et al. [12] adapted EDSR [13] as the main design for the SR reconstruction network in TDAN. Liang et al. [14] utilized residual Swin Transformer blocks from SwinIR [15] in their proposed RVRT. Although these works have adapted SISR models, each method utilizes only one SISR model. Applying SISR techniques to the VSR models would require considerable effort and they may not perform as effectively as specialized VSR models.

Meanwhile, several VSR methods do not rely on SISR models. For instance, Xue et al. [16] proposed TOF, which estimates task-oriented flow to recover details in SR frames. Wang et al. [17] proposed SOF-VSR, which estimates HR optical flow from LR frames. SWRN [18] can be utilized in real time on a mobile device. However, the development of a VSR model without adapting SISR methods is very costly, as the model needs to capture both temporal and spatial information. Moreover, compared with SISR methods, they may be less effective in utilizing spatial information.

To alleviate the above issues, we propose a plug-and-play approach for adapting existing SISR models to the VSR task. Firstly, we summarize a common architecture of SISR models and provide a formal analysis of adaptation to achieve better effectiveness of different SISR models. Then, we present an adaptation method, which inserts a plug-and-play temporal feature extraction module into SISR models. Specifically, the temporal feature extraction module consists of three submodules. The spatial aggregation submodule aligns features extracted by the original SISR model. The alignment is performed based on the result of the offset estimation submodule. Then, the temporal aggregation submodule is applied to aggregate information extracted from all neighboring frames.

To evaluate the effectiveness of the proposed method, we adapt five representative SISR models, i.e., SRResNet [19], EDSR [13], RCAN [20], RDN [21], and SwinIR [15], and the evaluations are conducted on two popular benchmarks, i.e., Vid4 and SPMC-11. On the Vid4 benchmark, the VSR-adapted models achieve at least 1.26 dB and 0.067 improvements over original SISR models in terms of peak signal-to-noise ratio (PSNR) [22] and structural similarity index (SSIM) [23], respectively. On the SPMC benchmark, the VSR-adapted models achieve at least 1.16 dB and 0.036 gain over original SISR models in terms of PSNR and SSIM, respectively. Moreover, the VSR-adapted models surpassed the performance of state-of-the-art VSR models.

For this paper, the main contributions are as follows: (1) We propose a plug-and-play approach for adapting SISR models to the VSR task. Instead of adapting one SISR model, the proposed method is based on a common architecture of SISR models. (2) A plug-and-play temporal feature extraction module is introduced. Thus, the adapted model gains the capability to exploit temporal information. (3) Extensive experiments are conducted to evaluate its effectiveness.

## 2. Related Work

### 2.1. Single-Image Super-Resolution

The SISR problem is an ill-posed problem, and learning-based methods have significantly improved the performance in terms of accuracy [13,15,19,20,21,24,25] and speed [26,27,28,29]. In 2014, Dong et al. [30] introduced a learning-based model, namely SRCNN, into the SISR field. Inspired by ResNet [31], Ledig et al. [19] proposed SRResNet in 2017. SRResNet [19] accepts LR images directly and achieves high performance and increased efficiency. Kim et al. [13] improved the SRResNet by removing unnecessary batch normalization in residual blocks and expanding the number of parameters. In 2018, Zhang et al. [21] employed a densely connected architecture. All extracted features are fused to utilize hierarchical information. Subsequently, Zhang et al. [20] introduced the channel attention mechanism that adaptively weights features channel-wisely. In 2021, Liang et al. [15] proposed SwinIR by making use of the Transformer [32]. Additionally, SwinIR uses the Swin Transformer [33] variation, which is more appropriate for computer vision tasks. By appropriately employing convolution layers and Swin Transformer modules, SwinIR can capture local and global dependencies at the same time, resulting in SOTA performance.

### 2.2. Video Super-Resolution

In recent years, deep-learning-based models have been used to solve the VSR problem, and have become increasingly popular [9]. We roughly divide VSR models into two categories:

(1) Models adapting SISR models: Sajjadi et al. [34] proposed FRVSR, which takes EnhanceNet [35] as the subnetwork for SR reconstruction. Haris et al. [10] applied the iterative up- and downsampling technique [11] in RBPN. The representative deep learning SISR model, EDSR [13], is utilized by many VSR models. Tian et al. [12] applied a shallow version of EDSR [13] in TDAN. EDVR [36] and WAEN [37] both employed the residual block and upsampling module from EDSR [13] in the reconstruction module. Inspired by [12], Xu et al. [38] adapted EDSR as the reconstruction module. EGVSR [39] applied ESPCN [26] as the backbone for the SR net. The recently proposed RVRT [14] utilized the residual Swin Transformer block, which is proposed in SwinIR [15].

(2) Models without adapting SISR models: DUF [40] reconstructs SR frames by estimating upsampling filters and a residual image for high-frequency details. Kim et al. [41] employed 3D convolution to capture spatial–temporal nonlinear characteristics between LR and HR frames. Xue et al. [16] proposed a method, namely TOF. It learns a task-specific representation of motion. Wang et al. [17] proposed SOF-VSR, which estimates HR optical flow from LR frames. To better leverage the temporal information, TGA [42] introduced a hierarchical architecture. Recently, Chan et al. [43] proposed BasicVSR by investigating the essential components of VSR models. Liu et al. [44] applied spatial convolution packing to jointly exploit spatial–temporal features. For better fusing information from neighboring frames, Lee et al. [45] utilized both attention-based alignment and dilation-based alignment. Lian et al. [18] proposed SWRN to achieve real-time inference while producing superior performance.

Because VSR models have to capture both temporal and spatial information, proposing a VSR method requires more effort. Thus, many researchers turn to adapting SISR models. Based on SISR models, proposing a VSR method can focus on capturing temporal information. However, these models either utilize a SISR model as a subnet or adapt modules from a SISR model to extract features. Additionally, they may be less effective than those methods that do not adapt SISR methods. Our work proposed a plug-and-play approach to adapt SISR models to the VSR task. The proposed method works on different SISR models as it follows the common architecture of SISR models we have summarized. The spatial information and temporal information are both extracted in the proposed method.

## 3. Methodology

In this section, we first summarize the common architecture of SISR models. Then, we provide a formal analysis of adaptation. Following that, a general VSR adaptation method is proposed. Finally, we present a plug-and-play temporal feature extraction module.

### 3.1. Revisit of Single-Image Super-Resolution Models

**Figure 1:**
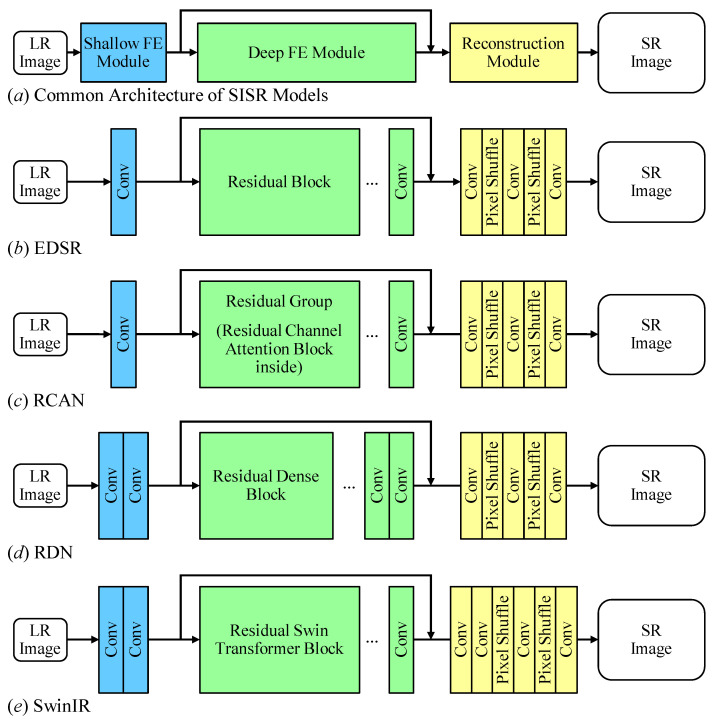
The architectures of typical SISR models.

For the effectiveness on different SISR models [13,15,19,20,21,46], we first summarize a common architecture, as shown in Figure 1. For simplicity, some operations such as element-wise addition and concatenation are omitted. As shown in Figure 1a, the common architecture of SISR models can be divided into three modules: shallow feature extraction (FE) module, deep FE module, and reconstruction module. Figure 1b–e illustrate the details of four SISR models. As one can see, the shallow FE module takes one LR image as input and extracts features by a few convolution layers. The deep FE module consists of several submodules or blocks, where advanced techniques, such as dense connection [21], channel attention [20], and self-attention [15], are applied. Thus, the deep FE module is where the key novelty of SISR models lies. Finally, the features from the deep FE module are fed to the reconstruction module to produce the SR image.

Thus, given an LR image $y\in R^{H\times W\times 3}$, these SISR models can be generalized using the following representation:

(1) $$x={Method}_{SISR}\left(y\right),$$

where ${Method}_{SISR}\left(\cdot \right)$ is the SISR model. $x\in R^{sH\times sW\times 3}$ represents the SR result with upscale factor *s*. *H* and *W* denote the height and width of LR image, respectively. According to the common architecture of SISR models, Equation (1) can be expanded as

(2) $$x=Recons({FE}_{deep}\left({FE}_{shallow}\left(y\right)\right)+{FE}_{shallow}\left(y\right)),$$

where the shallow and deep FE modules are noted as ${FE}_{shallow}\left(\cdot \right)$ and ${FE}_{deep}\left(\cdot \right)$, respectively. The reconstruction module is denoted as Recons(·).

Different from the SISR problem, the VSR methods have to exploit both spatial and temporal information. Thus, we make use of sliding window framework [12] to capture temporal dependency. Given consecutive 2n+1 LR frames $Y=\{y_{t-n},\cdots y_{t-1},y_{t},y_{t+1},\cdots y_{t+n}\}$, the representation of VSR models is formulated as

(3) $$x_{t}={Method}_{VSR}\left(Y\right),$$

where the VSR method is ${Method}_{VSR}\left(\cdot \right)$. $x_{t}$ represents the reconstructed SR frame, the frame index of which is *t*.

Note that the main difference between Equations (1) and (3) is the input, and Equation (2) is an expanded representation of Equation (1). In order to adapt existing SISR models to the VSR task, a straightforward method is to modify the shallow FE module. Then, the adapted model can be represented as

(4) $$x_{t}=Recons\left({FE}_{deep}\left({FE}_{shallow}^{\prime }\left(Y\right)\right)+{FE}_{shallow}^{\prime }\left(Y\right)\right),$$

where ${FE}_{shallow}^{\prime }\left(\cdot \right)$ is the modified shallow FE module.

### 3.2. Proposed Video Super-Resolution Adaptation Method

**Figure 2:**
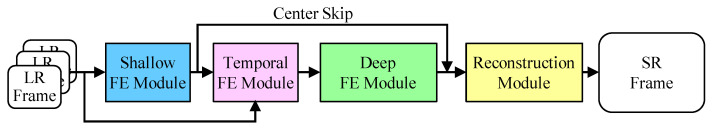
The Architecture of Proposed General VSR-Adapted Models.

According to the analysis in Section 3.1, we propose a general method to easily adapt SISR models to the VSR task. As shown in Figure 2, the architecture of the proposed VSR-adapted models consists of 4 modules. Firstly, the VSR-adapted model applies the shallow FE module ${FE}_{shallow}\left(\cdot \right)$ to obtain low-level features $F_{s,i}\in R^{H\times W\times C}$ for each LR frame $y_{i}$. The subscript ${}_{i}$ represents the relative index of the center frame. The center frame is denoted as ${}_{0}$, and *C* stands for the number of channels in a feature. The shallow feature of center frame $F_{s,0}$ is skip-connected to the output of the deep FE module with element-wise addition for global residual leaning. Secondly, the temporal FE module ${FE}_{temporal}\left(\cdot \right)$ is employed to exploit spatial–temporal information. It takes LR frames to estimate the offsets of pixels. It also takes shallow features which will be spatially aggregated based on the offsets. In order to enable the deep FE module to leverage information from all LR frames, spatial-aggregated features are temporally aggregated in the temporal FE module. Thirdly, the deep FE module ${FE}_{deep}\left(\cdot \right)$ is responsible for estimating accurate residual features with advanced techniques. Finally, the reconstruction module Recons(·) upsamples features with specific scale factors and produces SR frames. The architecture can be represented as

(5) $$F_{s,i}={FE}_{shallow}\left(y_{i}\right),$$

(6) $$F_{T}={FE}_{temporal}\left(F_{s,-n},\cdots ,F_{s,0},\cdots ,F_{s,n},y_{-n},\cdots ,y_{0},\cdots ,y_{n}\right),$$

(7) $$x_{0}=Recons\left({FE}_{deep}\left(F_{T}\right)+F_{s,0}\right),$$

where *i* denotes the relative index of the target frame, ranging from −n to *n*. The temporal feature $F_{T}\in R^{H\times W\times C}$ is the output of temporal FE module.

For adapting different SISR models, the proposed method maintains the shallow FE module, deep FE module, and reconstruction module unmodified. Furthermore, we employ the temporal feature extraction module between the shallow FE module and the deep FE module in accordance with accuracy and latency concerns.

From an accuracy perspective, the main difference between an input LR frame and its ground truth HR frame is the high-frequency content. Thus, the better the residual feature that is extracted, the better the achieved performance. The proposed architecture takes advantage of the deep FE module, where the key novelties of SISR models lie [46]. Further, with the information from neighboring frames, the deep FE module is able to extract more accurate features for reconstruction. Thus, the temporal FE module is employed before deep FE module.

From a latency perspective, the temporal FE module aggregates the features extracted from all input frames. It requires previous modules to complete their processing for each frame. To minimize the overall computation time, the proposed temporal FE module is employed after shallow FE module because its relatively small number of layers has a negligible impact on inference latency.

### 3.3. Plug-and-Play Temporal Feature Extraction Module

**Figure 3:**
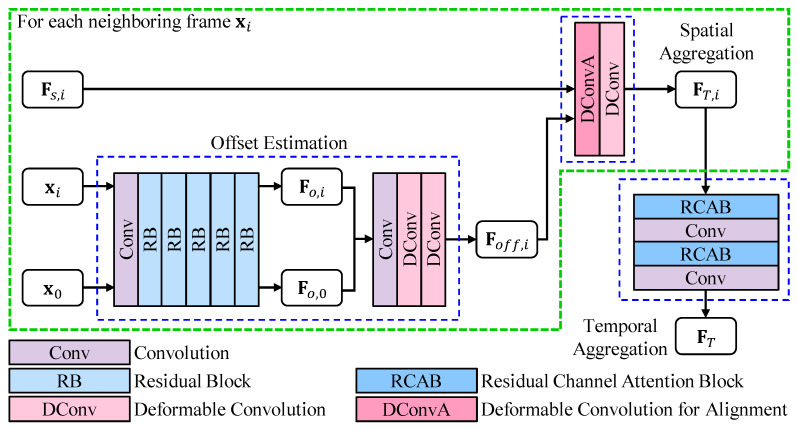
The Temporal Feature Extraction Module.

In order to exploit spatial–temporal information, the temporal FE module is proposed. The detailed architecture is illustrated in Figure 3, which consists of three submodules, i.e., offset estimation, spatial aggregation, and temporal aggregation.

The offset estimation submodule takes the center LR frame $y_{0}$ and each neighboring frame $y_{i}$ as inputs. The intermediate feature extraction is performed by a convolution layer and five residual blocks, and the parameters are shared across all input LR frames. The intermediate features are noted as $F_{o,i}\in R^{H\times W\times C}$. The offset feature $F_{off,i}\in R^{H\times W\times C}$ is estimated from the intermediate feature $F_{o,0}$ and $F_{o,i}$ using a convolution layer and two deformable convolution layers. The offset estimation submodule can be formulated as

(8) $$F_{o,i}={RB}_{5}\left(\cdots {RB}_{1}\left({Conv}_{1}\left(y_{i}\right)\right)\cdots \right),$$

(9) $$F_{off,i}={DConv}_{2}\left({DConv}_{1}\left({Conv}_{2}\left(CAT\left(F_{o,i},F_{o,0}\right)\right)\right)\right),$$

where RB(·) is residual block. Conv(·) and DConv(·) are convolution and deformable convolution, respectively. The concatenation is denoted as CAT(·).

The shallow feature $F_{s,i}$ and the estimated offset $F_{off,i}$ are then fed into the spatial aggregation submodule. Here, a variation of deformable convolution is used to extract features $F_{s,i}$, which takes $F_{off,i}$ for offset. This allows the offset feature $F_{off,i}$ to guide the alignment in the spatial aggregation submodule. Another deformable convolution is applied for refinement, resulting in output feature $F_{T,i}\in R^{H\times W\times C}$. The spatial aggregation submodule can be given by

(10) $$F_{T,i}={DConv}_{3}\left(DConvA(F_{s,i},F_{off,i})\right),$$

where DConvA(·,·) is the variation of deformable convolution. The variation of deformable convolution DConvA(·,·) takes the first input for feature extraction and the second input for offset.

After spatial aggregation, the temporal aggregation submodule fuses these spatial-aggregated features $F_{T,-n}\cdots F_{T,n}$. For fusing a feature with (2n+1)×C channels, a simple convolution layer is not sufficient. Therefore, a residual channel attention block [20] is employed to adaptively weight these features channel-wise. A convolution layer for channel reduction is then applied. The channel shrinkage is performed in two steps to minimize information loss: first reducing to twice the SISR features’ channels and then reducing to once. The temporal aggregation submodule can be represented as

(11) $$F_{T}={Conv}_{4}({RCAB}_{2}\left({Conv}_{3}\left({RCAB}_{1}\left(CAT\left(F_{T,-n},\cdots ,F_{T,n}\right)\right)\right)\right),$$

where $RCAB_{1}\left(\cdot \right)$ and $RCAB_{2}\left(\cdot \right)$ are residual channel attention blocks. The number of channels of the features output by ${Conv}_{3}\left(\cdot \right)$ and ${Conv}_{4}\left(\cdot \right)$ is 2×C and *C*, respectively. The temporal-aggregated feature is $F_{T}\in R^{H\times W\times C}$.

Overall, the spatial aggregation aligns neighboring features based on the result of the offset estimation submodule. Then, the temporal aggregation submodule fuses the spatial-aggregated features, resulting in an output containing information from all input LR frames. Finally, the plug-and-play module extracts feature $F_{T}$, which contains spatial–temporal information from all input frames. Further, we summarize the detailed algorithm of the VSR-adapted method with plug-and-play temporal feature extraction module in Algorithm 1. For easy understanding, we divided the loop into multiple ones.
**Algorithm 1:** Video Super-Resolution with SISR Model and Plug-and-Play Temporal Feature Extraction Module.
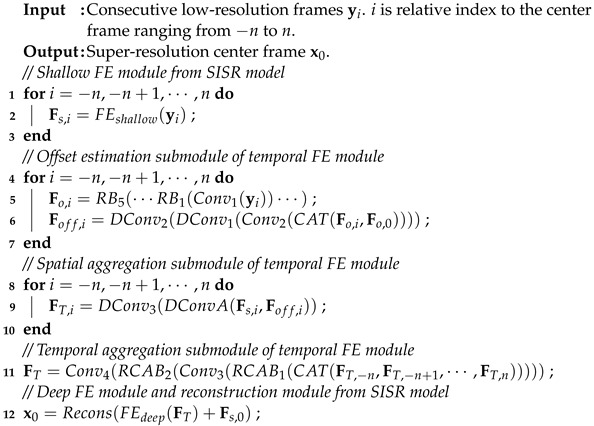


## 4. Experiment

### 4.1. Datasets

Following previous studies [12,16,47], we utilized the widely used Vimeo90K dataset for training. This dataset includes videos with different scenarios, such as moving objects, camera motion, and complex scene structures. It consists of 90,000 video clips with a resolution of 448×256. As per the official split, we use 64,612 video clips for training. The HR frames of these videos were used as the ground truth. For training, we randomly cropped these HR frames to patches with the size of 256×256, and these patches were bicubically downsampled to the size of 64×64 using the Matlab function *imresize*. We randomly flipped and rotated the data during training.

For testing, we evaluated the effectiveness of our proposed model on two public benchmarks, i.e., the Vid4 [48] and SPMC-11 [47]. The quantitative metrics were PSNR [22] and SSIM [23], computed in the luminance (Y) channel. We also cropped 8 pixels near the image boundary, similar to the previous approach [12].

### 4.2. Implementation Details

To evaluate the proposed method, we employed it on five representative SISR models: (1) SRResNet [19] is the generator model in SRGAN. (2) EDSR [13] is a representative SISR model. (3) RCAN [20] makes use of channel attention. (4) RDN [21] has the advantage of a dense connection. (5) SwinIR [15] introduces Swin Transformer [33]. For SISR models, we generated SR videos frame by frame.

In our implementation of SRResNet [19], we removed all batch norm layers. We used the EDSR baseline [13] with a feature channel count and block count of 64 and 16, respectively. For SwnIR [15], the LR patch size was 48×48, and the GT patch size was 192×192. We used a smaller patch size for SwinIR for lower memory consumption. The batch size for training all models was 16. We empirically set n=2, indicating that a VSR-adapted model takes five frames as input. For SISR models, the number of input frames was one. Each SISR model and its VSR-adapted model were trained from scratch using the same setting except for the number of input frames.

We used the mean square error (MSE) as the loss function, defined as $Loss={∥HR-SR∥}^{2}$. The parameters were updated using the Adam optimizer [49] with β1=0.9 and β2=0.99. The learning rate was initialized as $1\times 10^{-4}$ and halved for every $1\times 10^{5}$ iterations. We trained the models for $3\times 10^{5}$ iterations. All experiments were implemented in Pytorch and ran on a server with NVIDIA GPUs.

### 4.3. Effectiveness on Different Single-Image Super-Resolution Models

**Figure 4:**
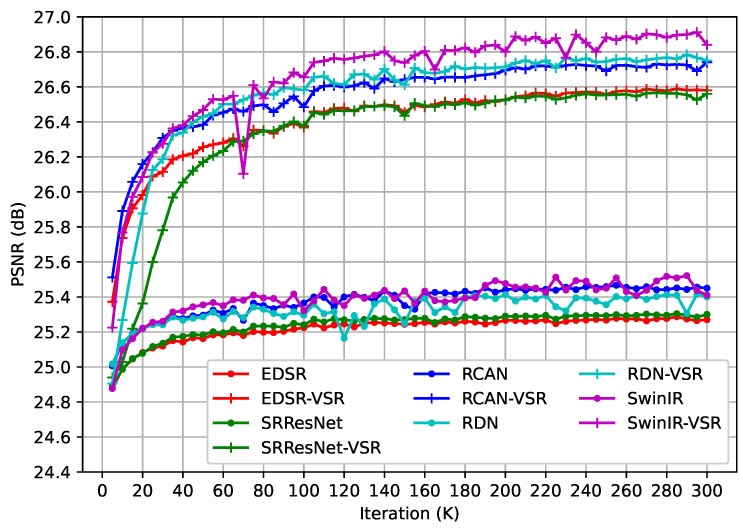
The PSNR Curve on Vid4 Benchmark During Training.

To evaluate the effectiveness of the proposed method, we conducted experiments on five representative SISR models. Table 1 displays the quantitative results on two popular benchmarks. The PSNR and SSIM metrics of VSR-adapted models improved by at least 1.16 dB and 0.036, respectively. It demonstrates that the proposed method works effectively on various SISR models. Moreover, the performance of the VSR-adapted models is positively correlated with the capacity of the original models. In the SISR task, EDSR [13] is better than SRResNet [19] but underperforms RCAN [20] and RDN [21]. The performance of RCAN and RDN is on par, and SwinIR [15] has the best performance. As shown in Table 1, the VSR-adapted models exhibit similar trends. We use the suffix “-VSR” to represent the VSR-adapted models. The performances of SRResNet-VSR and EDSR-VSR are weaker than those of RCAN-VSR and RDN-VSR, and SwinIR-VSR achieves the best results on both benchmarks. Moreover, we computed the PSNR metric on the Vid4 benchmark during training. As illustrated in Figure 4, the VSR-adapted models benefit from the information aggregated from neighboring frames, and they performed better in the early iterations during training. Thus, the proposed method is effective on different SISR models, and the plug-and-play temporal feature extraction module enables the VSR-adapted models to exploit spatial and temporal information.

**Figure 5:**
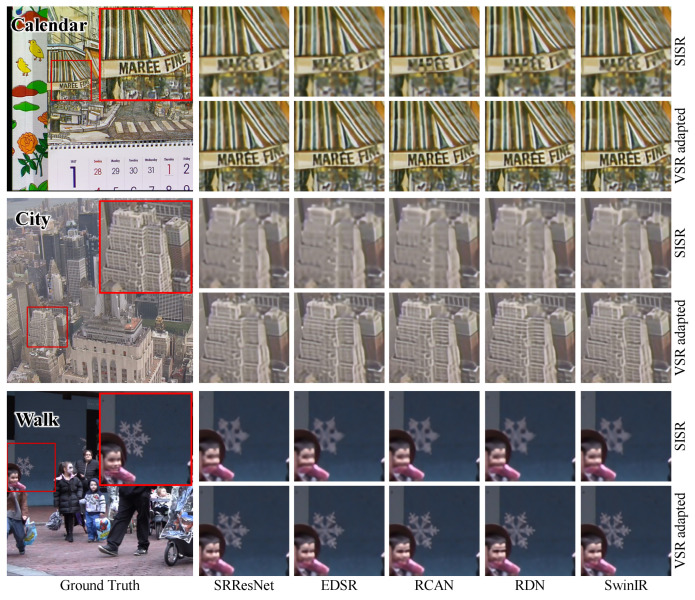
The Qualitative Comparison of SISR Models and Corresponding VSR Adaptations on Vid4 Benchmark.

**Figure 6:**
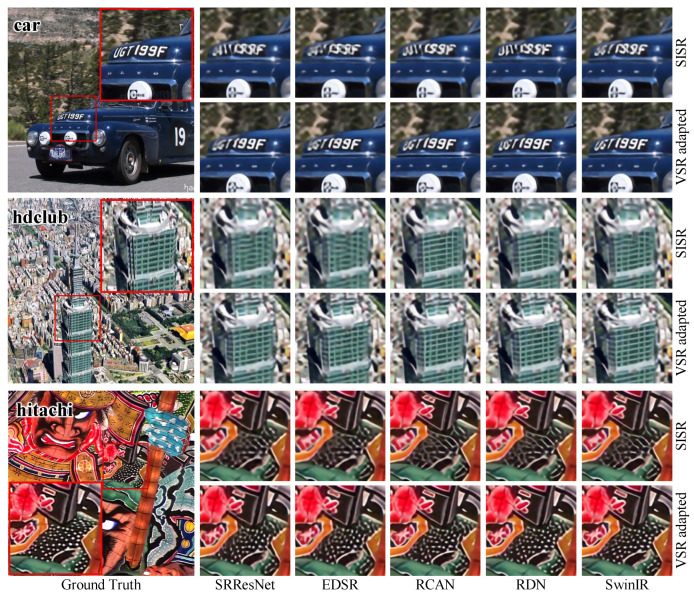
The Qualitative Comparison of SISR Models and Corresponding VSR Adaptations on SPMC-11 Benchmark.

Further, we visualized the results of the Vid4 and SPMC-11 benchmarks for qualitative comparison. Several processed frames are shown in Figure 5 and Figure 6. We can observe that the VSR-adapted models provide visually appealing results. By contrast, the original SISR models produce blurry SR frames and incorrect textures. Overall, the VSR-adapted models reconstruct results with clearer text, richer textures, and fewer artifacts. Among the results of the VSR-adapted models, SRResNet-VSR and EDSR-VSR produce more artifacts than other VSR-adapted models. This is consistent with the capabilities of original SISR models.

### 4.4. Comparisons with State-of-the-Art Methods

We compared these VSR-adapted models with 10 state-of-the-art VSR algorithms, i.e., STAN [50], EGVSR [39], TOFlow [16], STMN [51], SOF-VSR [17], ST-CNN [44], TDAN [12], D3Dnet [47], FRVSR [34], and WAEN [37]. Table 2 shows the quantitative metrics on the Vid4 and SPMC-11 benchmarks. The values with ^†^ are reported in [47]. As shown in Table 2, the VSR-adapted models achieve competitive performance on both Vid4 and SPMC-11 benchmarks. All VSR-adapted models perform better than D3Dnet. Compared with D3Dnet, the SRResNet-VSR and EDSR-VSR achieve comparative performance. The performances achieved by RCAN-VSR and RDN-VSR are between FRVSR and WAEN. Among them, the SwinIR-VSR outperforms all models in terms of PSNR metrics.

**Figure 7:**
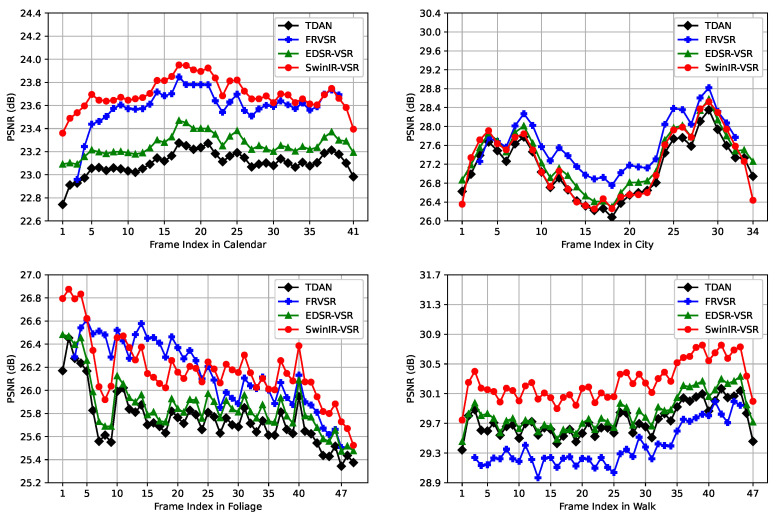
The PSNR curve of VSR models on Vid4 benchmark.

For a finer quantitative comparison on the Vid4 benchmark, we illustrate the PSNR metric of each frame in Figure 7. For simplicity, we select four models, i.e., TDAN [12], FRVSR [34], EDSR-VSR, and SwinIR-VSR. Compared with TDAN, the EDSR-VSR achieves similar performance. Note that the first two and last two frames show a greater difference between TDAN and EDSR-VSR. Because there is less neighboring information for VSR models to exploit, the VSR models exhibit poor performance at the beginning and end of a video. Compared with FRVSR, the SwinIR-VSR achieved better performance on the *Calendar* and *Walk*. As the frame index increases on the *Calendar*, the gap between SwinIR-VSR and FRVSR becomes smaller. Additionally, the performance of SwinIR-VSR is lower than that of FRVSR after the first five frames on the *City*. This is because the SwinIR-VSR makes use of neighboring frames in a sliding window scheme while the FRVSR utilizes them in a recurrent scheme.

**Figure 8:**
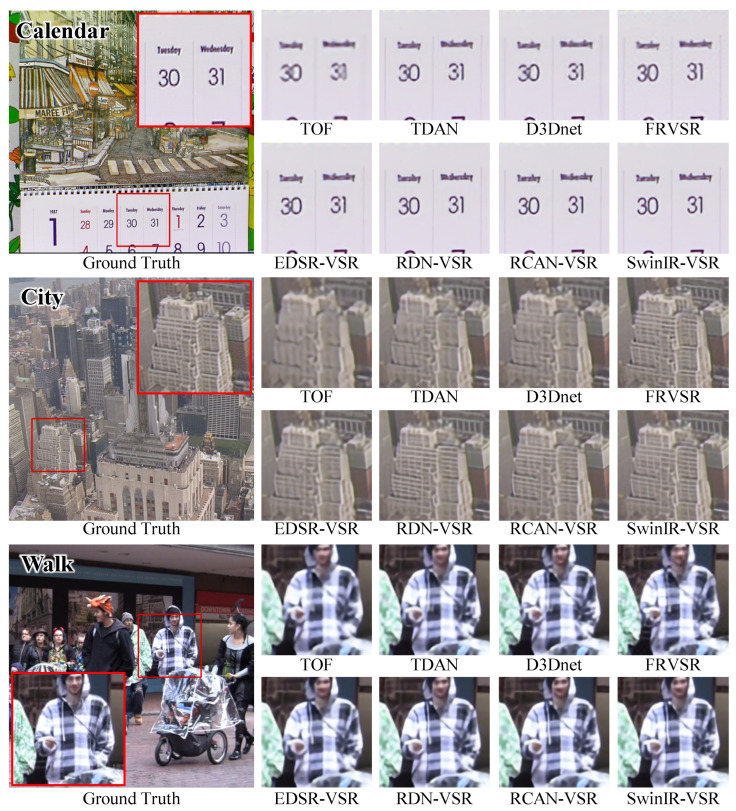
Qualitative Comparison of VSR Models on Vid4 Benchmark.

**Figure 9:**
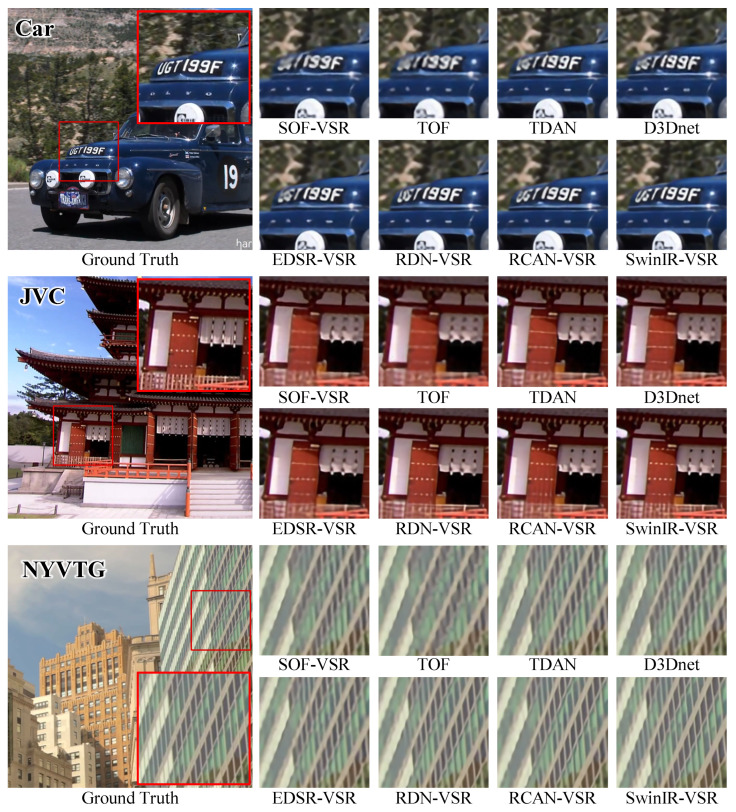
Qualitative Comparison of VSR Models on SPMC-11 Benchmark.

For a qualitative comparison, we compared the VSR-adapted models to SOF-VSR [17], TOF [16], TDAN [12], D3Dnet [47], and FRVSR [34]. As shown in Figure 8, the VSR-adapted models reconstruct visually attractive results. The text on the *Calendar* is now easier to read and the details of the *City* are clearer. Additionally, the clothes in the *Walk* image are more recognizable. Moreover, we observed similar trends in the SPMC-11 benchmark, as illustrated in Figure 9. The quality of the reconstructed results of EDSR-VSR is equivalent to that of the compared methods. The RDN-VSR and RCAN-VSR provide results with better quality. The result of SwinIR-VSR has the least artifacts.

### 4.5. Comparisons of Temporal Consistency

**Figure 10:**
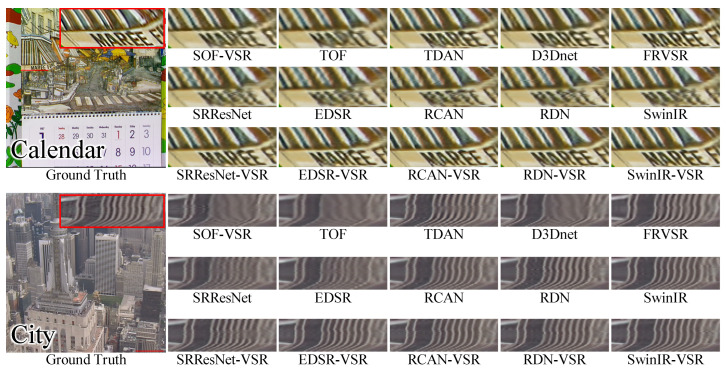
Qualitative Comparison of Temporal Profile on Vid4 Benchmark.

To evaluate the temporal consistency of the proposed method, we generated temporal profiles according to [34] for visualization. As shown in Figure 10, the positions of temporal profiles are highlighted with red lines. The heights of temporal profiles vary due to the video length. As shown in the *Calendar*, the temporal profiles demonstrate that the original SISR models perform poorly because they are unable to capture temporal information. By contrast, the VSR methods and VSR-adapted models produce results with fewer artifacts. However, inappropriate aggregation of temporal information can lead to degraded results. As illustrated in the *City*, the original SISR models and our VSR-adapted models exhibit better temporal consistency than VSR models.

### 4.6. Ablation Study

We used EDSR [13] as the baseline in the ablation study to evaluate the effectiveness of the proposed temporal feature extraction module, which consists of offset estimation, spatial aggregation, and temporal aggregation submodules. We evaluated three models to determine the effectiveness of each submodule. The first variation is denoted as Model 1. We fed shallow features from neighboring frames to the spatial aggregation submodule without the support of the offset estimation submodule. The neighboring features were then fused with a convolution using a 1×1 kernel. Model 2 is referred to as the second variation. We introduced the offset estimation submodule, which makes use of the center frame and neighboring frames to guide the spatial aggregation. The third variation, denoted as EDSR-VSR, combines all the components, including channel attention and progressive channel shrinking.

Table 3 indicates that relying solely on the spatial aggregation submodule does not lead to performance improvement. However, with the support of the offset estimation submodule, there is a significant performance improvement. Furthermore, the temporal aggregation submodule further improved the performance. Three submodules play an irreplaceable role in our presented temporal feature extraction module.

To evaluate the efficiency of the proposed method, we conducted a comparison on the Vid4 benchmark. We evaluated three models, i.e., EDSR [13], EDSR-VSR, and EDSR-VSR 2. The EDSR-VSR 2 employs the temporal feature extraction module after the deep feature extraction module. Table 4 shows the performance and average latency of inference. As we can see, the EDSR-VSR is about 1.6× faster than the EDSR-VSR 2. Although the EDSR-VSR is slower than EDSR [13], it reaches 24 frames per second. Specifically, we analyzed the latency of each part of EDSR-VSR. Overall, 0.89% of the latency is consumed by the shallow feature extraction module from the SISR model. The subsequent offset estimation submodule, spatial aggregation submodule, and temporal aggregation submodule occupied 21.25%, 39.99%, and 15.21% of the latency, respectively. Additionally, 22.66% of the time is spent on the deep feature extraction and reconstruction module from the SISR model. Note that the temporal feature extraction module has to process all input frames, so each submodule takes a longer time to complete the computation. Thus, the proposed method balances the accuracy and latency.

## 5. Discussion and Limitation

The proposed method builds a bridge between the SISR model and the VSR model. We revisited many SISR models and summarized a common architecture of SISR models. The proposed method leverages the inherent similarities and differences between the two tasks, and the plug-and-play temporal feature extraction module is presented to allow the VSR-adapted model to utilize information from neighboring frames. We applied it to five representative SISR models to evaluate our method, including a generator of GAN [19], three representative SISR models [13,20,21], and a Transformer-based model [15]. Compared with state-of-the-art VSR models, our VSR-adapted models achieve competitive performance.

There are several strong points of the proposed method. Firstly, the proposed architecture of VSR-adapted models provides a novel scheme to develop VSR models. As long as a SISR model follows the common architecture, it can be easily adapted to a VSR model. It reduces the delay of applications of new SISR technologies. Secondly, with the development of VSR, better temporal feature extraction techniques will be proposed, leading to better VSR performance. It divides the development of the VSR model into two independent tasks. Thirdly, the plug-and-play characteristic enables a single model to perform both SISR and VSR tasks.

**Figure 11:**
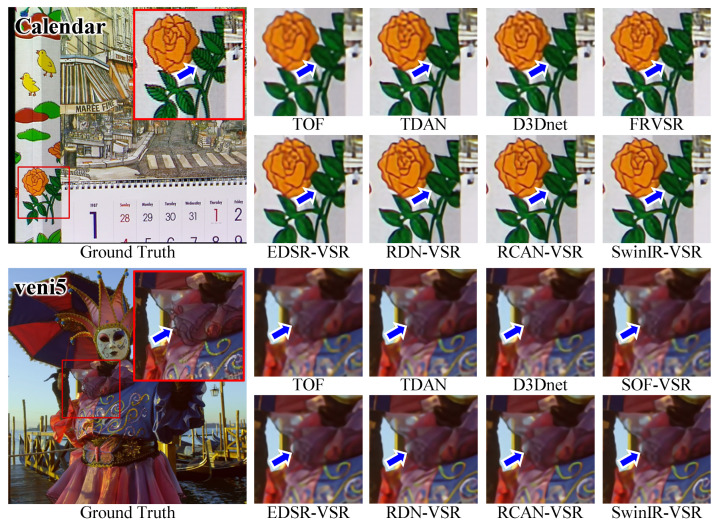
The Qualitative Comparison of Details in Low-Contrast Areas.

Although the VSR-adapted models show promising results, we observed some failure cases in experiments. As illustrated in Figure 11, these models fail to recover tiny details. In these cases, the contrast is low in the ground truth, and the contrast is further reduced in LR frames, making SR reconstruction very challenging. Furthermore, all VSR-adapted models fail to provide clear results.

## 6. Conclusions

In this paper, we propose a method for adapting SISR models to the VSR task. For effectiveness on various SISR models, we summarize the common architecture of SISR models. The VSR-adapted models leverage the capability of SISR models to learn the mapping between LR and HR images. Then, the proposed plug-and-play temporal feature extraction module allows VSR-adapted models to access spatial–temporal information. Thus, the performance in the VSR task is improved by the incorporation of the SISR model and the temporal feature extraction module. The experiments on several SISR models and benchmarks show that VSR-adapted models surpass the original SISR models. The achieved performance is positively related to the capacity of SISR models, indicating the effectiveness of the proposed method. Further, the VSR-adapted models achieved better results than the SOTA VSR models. In the future, we plan to solve the problem of poor performance in low-contrast areas.

## Figures and Tables

**Table 1:** Quantitative Comparison of SISR Models and VSR-Adapted Models on Vid4 and SPMC-11. The best results are in bold.

		Original		VSR Adapted	
Benchmark	Method	PSNR	SSIM	PSNR	SSIM
	SRResNet [19]	25.30	0.728	26.56	0.797
	EDSR [13]	25.27	0.726	26.58	0.798
Vid4	RCAN [20]	**25.45**	0.737	26.74	0.804
	RDN [21]	25.40	0.734	26.75	0.806
	SwinIR [15]	25.41	**0.738**	**26.84**	**0.811**
	SRResNet [19]	27.92	0.815	29.16	0.853
	EDSR [13]	27.85	0.813	29.14	0.853
SPMC-11	RCAN [20]	28.32	0.823	29.48	0.859
	RDN [21]	28.24	0.821	29.55	0.862
	SwinIR [15]	**28.46**	**0.826**	**29.74**	**0.866**

**Table 2:** Quantitative comparison of Vid4 and SPMC-11. The best results are in bold. The values with ^†^ are reported in [47].

	Vid4		SPMC-11	
Method	PSNR (dB)	SSIM	PSNR (dB)	SSIM
STAN [50]	25.58	0.743	—	—
EGVSR [39]	25.88	0.800	—	—
TOFlow [16]	25.90	0.765	—	—
STMN [51]	25.90	0.788	—	—
SOF-VSR [17]	26.02	0.772	28.21 ${}^{†}$	0.832 ${}^{†}$
ST-CNN [44]	26.12	**0.823**	—	—
TDAN [12]	26.42	0.789	28.51 ${}^{†}$	0.841 ${}^{†}$
D3Dnet [47]	26.52	0.799	28.78	0.851
FRVSR [34]	26.69	0.822	—	—
WAEN [37]	26.79	—	—	—
SRResNet-VSR	26.56	0.797	29.16	0.853
EDSR-VSR	26.58	0.798	29.14	0.853
RCAN-VSR	26.74	0.804	29.48	0.859
RDN-VSR	26.75	0.806	29.55	0.862
SwinIR-VSR	**26.84**	0.811	**29.74**	**0.866**

**Table 3:** The Effectiveness of Each Component in Temporal Feature Extraction Module.

Dataset	Model	Spatial Aggregation	Offset Estimation	Temporal Aggregation	PSNR (dB)	SSIM
	EDSR [13]	✗	✗	✗	25.27	0.726
	Model 1	✓	✗	✗	25.31	0.725
Vid4	Model 2	✓	✓	✗	26.49	0.793
	EDSR-VSR	✓	✓	✓	26.58	0.798
	EDSR [13]	✗	✗	✗	27.85	0.813
	Model 1	✓	✗	✗	27.88	0.813
SPMC-11	Model 2	✓	✓	✗	28.97	0.849
	EDSR-VSR	✓	✓	✓	29.14	0.853

**Table 4:** The Efficiency of Proposed Method on Vid4 Benchmark.

	EDSR [13]	EDSR-VSR	EDSR-VSR 2
PSNR (dB)	25.27	26.58	26.61
SSIM	0.726	0.798	0.798
Latency (ms)	9.872	41.543	65.003

## Data Availability

The public data used in this work are listed here: Vimeo90k http://tof-low.csail.mit.edu/ (accessed on 12 December 2022), Vid4 https://drive.google.com/file/d/1ZuvNNLgR85TV_whJoH-M7uVb-XW1y70DW/view?usp=sharing (accessed on 12 December 2022), and SPMC-11 https://pan.baidu.com/s/1PK-ZeTo8HVklHU5Pe26qUtw (accessed on 12 December 2022) (Code: 4l5r).
